# Use of Johan forceps as endoloop pushers for laparoscopic appendicectomy

**DOI:** 10.1308/003588412X13373405387050f

**Published:** 2012-10

**Authors:** K Siddique, N Siddiqi, P Sedman

**Affiliations:** ^1^Wirral University Teaching Hospital NHS Foundation Trust,UK; ^2^Hull and East Yorkshire Hospitals NHS Trust,UK

## BACKGROUND

Laparoscopic appendicectomy is one of the most common surgical procedures and is often performed out of hours. Securing the base of the appendix may be achieved either by stapling or by use of a pre-formed endoloop.^1^ Stapling is an expensive option and mandates the use of a 12mm trocar. Pre-formed single-use endoloops with single-use (often integral) knot pushers may not always be accessible. We present an inexpensive alternative to the pre-formed endoloop and a technique to safely push the knot using universally available 5mm laparoscopic forceps.

## TECHNQIUE

**Figure 1:**
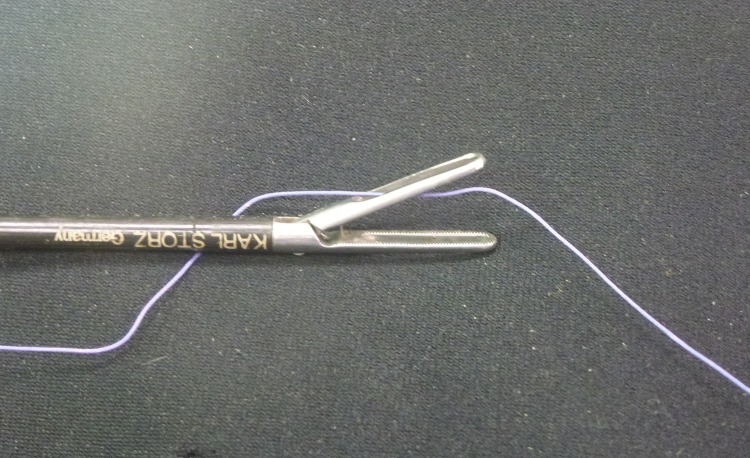
Long end of endoloop passed through the upper jaw of Johan forceps

**Figure 2:**
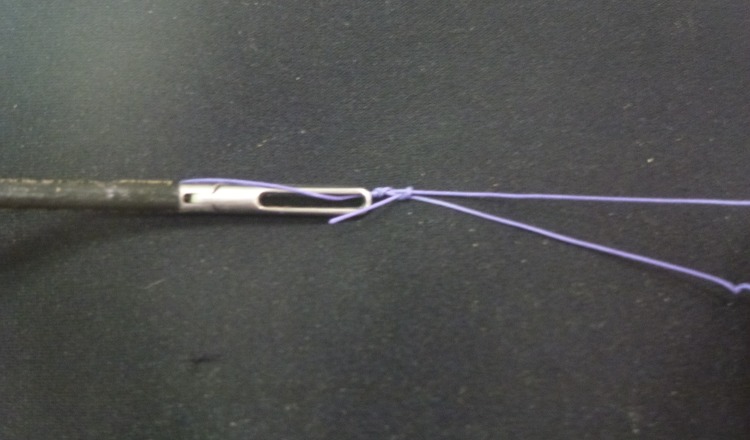
Tip of Johan forceps in close approximation with the knot

**Figure 3:**
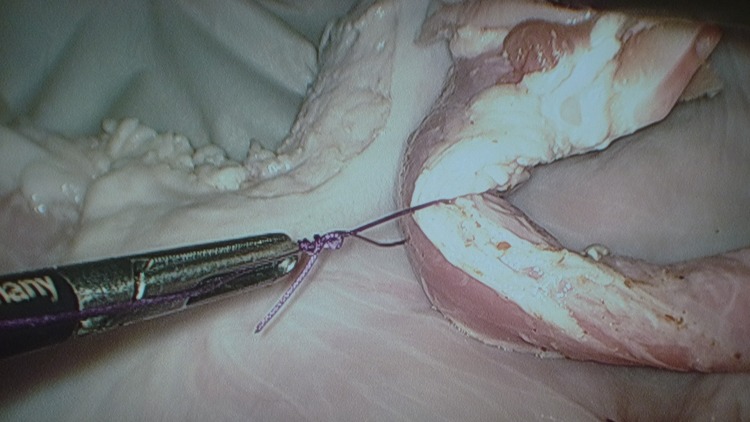
Johan forceps tip acting as a guide to help controlled placement of knot

An endoloop is created extracorporeally using a Roeder knot in a standard fashion.^2^ The standing end is kept long and passed through the fenestration of the mobile jaw of a Johan forceps (Fig 1). The jaws of the forceps are then loosely approximated behind the knot to act as the knot pusher (Fig 2) and this ensures smooth delivery of the ligature (Fig 3). Care is needed to ensure the jaws are not closed too tightly as the serrations may shred the suture material. We have found the optimal suture to be a size 1 Vicryl® suture (Ethicon Inc, Somerville, NJ, US), which balances knot security with thread robustness and strength. Monofilaments are an alternative.

**Figure 4:**
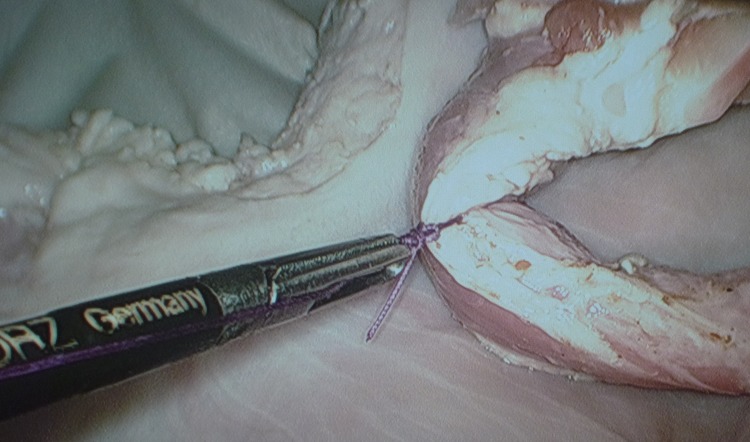
Secure and precise placement of knot at the desired site for ligature tightening

The advantage of passing the standing end through the fenestration as shown is that the knot is always easily retrieved should the forceps and standing end become misaligned during the pushing process. The technique is equally applicable to loops created extracorporeally and used in a lasso fashion for pedicle ligation (Fig 4) and for sutures passed intracorporeally but tied extracorporeally. In the latter case, greater care is required to define the standing end during the knot tying process.

## DISCUSSION

The self-created endoloops are reliable, safe and cost effective.^3^ The base of the appendix can be secured safely without the need for expensive commercial endoloops or a knot pusher, thereby significantly reducing costs. This technique is also adaptable for the creation of extracorporeal knots for ligation in continuity as might be desired for tying off an appendicular artery. Our technique has the added benefits of secure knot placement at the correct anatomical site with the help of Johan’s forceps, which has not been described previously.
